# Impact of case volume per year on flexible Ureteroscopy practice: an internet based survey

**DOI:** 10.1186/s12894-019-0568-z

**Published:** 2019-12-18

**Authors:** Omar Alhunaidi, Abdulrahman A. Ahmad, Ahmed R. EL-Nahas, Bader Akroof, Ali Alamiri, Feras Al-Ajrawi, Abdullatif Al-Terki, Mohamed El-Shazly

**Affiliations:** 1 Urology Department, Al-Amiri hospital, Kuwait City, Kuwait; 2 Urology Department, Adan hospital, Hadiya, Kuwait; 3 Urology and Nephrology Center, Mansoura University, Mansoura, Egypt; 4 Urology Department, Al-Farwaniya hospital, Sabah Al Nasser, Kuwait; 5 Urology Department, Menoufia University, Al Minufya, Egypt

**Keywords:** Flexible ureteroscopy, Survey, Stones, Ureteral access sheath, Flexible URS

## Abstract

**Background:**

To report current worldwide variation in techniques and clinical practice of flexible ureteroscopy (FURS) among endourologists of different case volumes per year.

**Methods:**

Two invitations to complete an internet survey were emailed to Endourological Society members. Some of survey questions asked about indications of using FURS for renal and upper ureteral stones. Others were concerned with clinical practice of FURS (such as preoperative stenting, use of ureteral access sheath (UAS) and safety guidewire, technique of Laser lithotripsy and fragment retrieval, and post-FURS stenting. Responders were distributed into two groups; high-volume (> 100 cases/year) and low-volume surgeons (< 100 cases/year) and data were compared between both groups.

**Results:**

Responses were received from 146 endourologists all over the world (62 high-volume and 84 low-volume). FURS for intrarenal stone > 20 mm was used by 61% of high-volume surgeons compared with 28.6% for low-volume (*P* < 0.001). Semirigid URS was used for upper ureteric stones in 68% among high-volume group and 82% in low-volume group (*P* = 0.044). UAS was used by 62% in low-volume group and 69% in high volume group (*P* = 0.516). Laser stone dusting was preferred by 63% in low-volume group versus 45% by high-volume (*P* = 0.031). More responders in low-volume group preferred to leave the stent for 6 weeks (*P* = 0.042).

**Conclusions:**

The use of FURS for treating upper tract calculi has expanded by high volume endourologists to include large renal stones > 20 mm. Low-volume surgeons prefer to use semi-rigid URS for treatment of upper ureteral stones, to apply Laser stone dusting and maintain ureteral stents for longer periods.

## Background

Treatment of upper urinary tract calculi has changed in recent years because of development of minimally invasive techniques such as flexible ureteroscopy (FURS) and miniaturized percutaneous nephrolithotomy (mini, ultra-mini and micro PCNL) [1]. Technological advances in FURS design (such as improved active tip deflection and digital image transfer), Laser lithotripsy techniques, advances in instruments as tipless baskets and ureteral access sheath (UAS) have resulted in increasing the utilization of FURS in treatment of renal and upper ureteric calculi. European Association of Urology (EAU) Guidelines recommended shock waves lithotripsy (SWL) and endourological for treatment of renal stones < 2 cm [2]. AUA guidelines recommended PCNL for treatment of large renal stones < 2 cm [3]. However, some experienced authors reported successful and safe use of FURS for treating larger stone burdens < 2 cm [4, 5].

The large variety of instruments used during FURS offered increased surgical efficiency, decreased frequency and severity of complications, and improved cost effectiveness. Many controversies in FURS indications, techniques and utilization of available instruments were addressed in previous surveys [6, 7]. However, the effect of case volume per year on selection of certain indications, utilization of specific techniques and preference of instruments was reported in only one study that involved only European urologists [7]. High-volume URS cases per year was proved to result in better outcomes (such as shorter operative time, better stone free rates, shorter hospital stay, less need for retreatment, fewer and less severe complications) when compared with lower case-volume [8].

This study was conducted to report current worldwide variation in techniques and clinical practice of FURS among endourologists of different FURS workload per year.

## Methods

### Study design

An email invitation was sent to Endourological society members to answer an anonymous online survey in June 2017. A reminder email was sent after one month. The survey was conducted through SurveyMonkey® system and responses were collected over three months. By accepting the invitation to complete the online survey, all participants provided their informed consent to take part in the study. This study design did not require ethical approval because no patients were included.

### Survey

The online questionnaire contained 14 questions (three questions asked about country, period of doing FURS and number of FURS per year, two asked about using FURS for upper ureteral and large renal stones and 9 questions were concerned with clinical practice of FURS (such as preoperative stenting and urine culture, use of ureteral access sheath (UAS) and safety guidewire, type of FURS (fiberoptic or digital), technique of Laser lithotripsy and fragment retrieval, post-FURS stenting indications and period).

### Participants

Responders were divided into two groups based on number of cases per year, high-volume surgeon with > 100 case/year and low-volume surgeon < 100 case/year [6]. FURS technical details and practice were compared between the two groups.

### Statistical analysis

Statistical analysis was performed using SPSS V20 software (USA). All variables were categorical and were expressed as numbers and percentages. Chi-square test was used to compare the difference between both groups. *P* Value < 0.05 was considered statistically significant.

## Results

### Participants

The survey was answered by 146 responders of 1204 (12%) Endourological society members from 40 countries. They represent all continents (50 from North America, 42 from Europe, 29 from Asia, 16 from South America and 9 from Africa).

### Differences between high and low-volume Endourologists

Table 1 summarized the comparison between both groups. High-volume surgeons were practicing FURS for significantly longer periods than low-volume group (*P* < 0.001). FURS for intrarenal stones > 20 mm was used by 61.3% of high-volume group versus 28.6% of low-volume group (P < 0.001). Semi-Rigid ureteroscopy for upper ureteric stone was more frequently preferred by low-volume group (82%) in comparison with 68% in high-volume group (*P* = 0.044). Stone dusting was preferred by 63% by low-volume group versus 45% by high-volume (*P* = 0.031). Another significant difference was the period of post-URS stents as more responders in low-volume group preferred to leave the stent for 6 weeks (*P* = 0.042).

**Table 1:** Comparison between high and low-volume groups

Variable	Low-volume (< 100 cases/year) 84	High-volume (> 100 cases/year) 62	*P* value	Total 146
	N. (%)	N. (%)		
Years of doing FURS			< 0.001	
< 5 Years	23 (27.4)	6 (9.7)		29 (20)
5–10 Years	28 (33.3)	12 (19.3)		40 (27.3)
> 10 Years	33 (39.3)	44 (71)		77 (52.7)
FURS for stones >2 cm:			< 0.001	
Yes	24 (28.6)	38 (61.3)		62 (42.5)
No	60 (71.4)	24 (38.7)		84 (57.5)
Semi-Rigid URS for Upper			0.044	
Ureteric Stones:				
Yes	69 (82)	42 (67.7)		111 (76)
No	15 (18)	20 (32.3)		35 (24)
Pre-FURS Ureteric stent			0.492	
Yes	4 (4.8)	2 (3.2)		6 (4)
No	80 (95.2)	60 (96.8)		140 (96)
Preoperative Urine Culture:			0.331	
Yes	65 (77.4)	52 (84)		117 (80)
No	19 (22.6)	10 (16)		29 (20)
Type of FURS:			0.287	
Digital	28 (33.3)	26 (42)		54 (37)
Fiberoptic	56 (66.7)	36 (58)		92 (63)
Use of Safety Guidewire:			0.312	
No	5 (6)	8 (13)		13 (9)
In some cases	22 (26)	17 (27.3)		39 (26.6)
In all cases	57 (68)	37 (59.7)		94 (64.4)
Ureteral Access Sheath			0.516	
Yes	52 (62)	43 (69.4)		95 (65)
No	3 (3.5)	3 (4.8)		6 (4.2)
In prestented cases	29 (34.5%)	16 (25.8)		45 (30.8)
Laser Lithotripsy			0.031	
Dusting	53 (63)	28 (45.2)		81 (55.5)
Fragmentation	31 (37)	34 (54.8)		65 (44.5)
Fragments retrieval			0.485	
No Retrieval	22 (26.2)	12 (19.4)		34 (23.3)
Basket	56 (66.7)	47 (75.8)		103 (70.5)
Forceps	6 (7.1)	3 (4.8)		9 (6.2)
Postoperative Stent			0.539	
All cases	49 (58.3)	33 (53.2)		82 (56.2)
Indicated cases	35 (41.7)	29 (46.8)		64 (43.8)
Period of stenting after ureteric injury:			0.042	
2 Weeks	12 (14.3)	12 (19.4)		24 (16.5)
4 weeks	52 (62)	45 (72.6)		97 (66.5)
6 weeks	20 (23.7)	5 (8)		25 (17)

### Similarities between high and low-volume Endourologists

Regardless of the case volume per year, most responders (96%) used FURS in non-stented ureters, 80% request preoperative urine culture, 76% were using semirigid URS for upper ureteric stones and 70% used a basket for retrieval of stone fragments after disintegration. Two third of responders routinely uses a safety guidewire and UAS during FURS (Table 1).

## Discussion

The superior results of surgeons with higher volume URS cases in comparison with others with lower volume cases was attributed to increasing surgical experience of the treating team (endourologist, nurses and technicians) [8]. In the present study, we reported the effect of case-volume per year on clinical practice and preference of certain instruments and surgical techniques of FURS.

High-volume responders in the present survey performed significantly more FURS for renal calculi > 20 mm despite that EAU and American Urological Association (AUA) guidelines [2, 3] that recommended PCNL as the first line for this stone size. However, high-volume endourologists used their experience to reach efficient outcomes [5, 8]. Moreover, we observed an increase in the overall preference of FURS for large renal stones among all responders (42%) in comparison with the survey conducted by Dauw et al. who reported 11% [6]. This may be explained by the increased experience of FURS surgical techniques and advances in Laser lithotripsy technology.

The second important difference was the preference of semirigid URS for treatment of upper ureteric calculi. Although high-volume endourologists used semirigid URS less frequently than low-volume group (68% vs 82%, *P* = 0.044). However, a considerable number of endourologists in each group preferred semirigid URS for treatment of upper ureteric stones. This may be attributed to financial causes in some countries, less experience in using FURS by some urologists (especially in low-volume group) or choosing to start with semirigid URS and if stone fragments escaped to the kidney, FURS can be used to retrieve them. AUA guidelines recommended that a FURS should be available when performing URS for proximal ureteric calculi [3]. Galal et al. and Karadag et al. compared both techniques for treatment of proximal ureteric calculi and reported that FURS offered significantly better stone free rate (*P* = 0.005) but rigid URS had significantly shorter operative time (P = 0.005) and both had comparable complication rates [9, 10].

The third difference in this survey was utilization of Laser dusting of stones by more endourologists in low-volume group (63% vs 45%, *P* = 0.031). High-volume endourologists prefer fragmentation and stones retrieval to achieve immediate stone free status in most patients, while low-volume surgeons prefer dusting and leaving gravels for spontaneous passage. Dusting uses low energy, high-frequency and long pulse duration setting to fragment stones into fine powder and small fragments that are left for spontaneous passage [11]. The stone free rates for fragmentation and basket retrieval were significantly better than dusting, but operative time was shorter for dusting [12, 13].

The use of UAS with FURS has been a controversial issue in urological literature. Advantages of UAS include ease of multiple passages of the scope, rapid stone extraction, higher stone free rates, low intrarenal pressure during prolonged procedures [14, 15]. Other reports showed no advantages of UAS for stone free rate or operative time, while the incidence of complications was higher with UAS [16, 17]. In the present survey, the use of UAS was practiced by 62 and 69% of responders in group 1 and 2 respectively (*P* = 0.516). It is obvious that about two thirds of endourologists of different levels of case load believe that the advantages of UAS outweigh its disadvantages. A similar result was reported from a large European survey where 70.7% used UAS [7].

When asking about the use of a safety guidewire, 68% of group 1 and 60% of group 2 indicated that they used it in all cases. In the past, use of a safety guidewire was highly advised to allow smooth access to the pelvicalyceal system and facilitates ureteral stent placement in case of ureteral or collecting system injury [18]. A study by Western Endourological stone consortium showed that the risks for FURS damage were significantly decreased when using a safety guidewire [19]. Currently; and with increased use of UAS; there are some reports of FURS without a safety guidewire [20, 21]. In the present study, it was observed that 13% of high-volume surgeons did not use a safety guidewire compared to 6% of the other group. These rates are more than the reported 1.9% in the previous survey by Dauw et al. [6] and we think that it is expected to increase in future.

Another controversial issue is the placement of ureteric stent either before or after FURS. The present survey showed that the majority endourologists in both groups follow the AUA guidelines [3] in omitting ureteral stent placement prior to FURS. For post-FURS stenting, the AUA guidelines recommended ureteric stent placement in certain indications such as ureteral injury, ureteric stricture, large stone size, incomplete stone fragmentation, solitary kidney or impaired renal function [3]. However, in the present survey 58% of group 1 and 53% of group 2 placed a stent in all cases after FURS. These rates are comparable to previously reported 64% by Dauw et al. [6] and slightly more than the 44% reported in the European survey [7].

Low response rate (12%) in comparison with 20% in Dauw et al. survey among Endourology Society members [6] was the major limitation of the present study. However, responders represented all regions around the world. This survey included specific group of urologists who are interested in endourology as primary subspecialty. Therefore, the results cannot be extrapolated to general urologists. On the other hand, this study showed the differences in clinical practice of FURS among endourologists of low versus high-case volume. This is highly valuable to know the current controversies, agreement or disagreements with international guidelines. The future implications include conducting randomized controlled studies to compare the safety and efficacy of controversial issues involving FURS indications, instruments and clinical practice.

## Conclusions

The use of FURS for treating upper tract calculi has expanded by high-volume endourologists to include large renal stones > 20 mm. Low-volume surgeons prefer to use semi-rigid URS for treatment of upper ureteral stones, to apply Laser stone dusting and maintain ureteral stents for longer periods. Ureteral access sheath is commonly used whatever the case-volume per year.

## Data Availability

All data are available for review on request.

## Abbreviations

AUA: American Urological Association
EAU: European Association of Urology
FURS: Flexible ureteroscopy
PCNL: Percutaneous nephrolithotomy
UAS: Ureteral access sheath
URS: Ureteroscopy
